# The Importance of Positive Psychological Strengths in Well-Being and Adjustment of Romanian Emerging Adults: A Pattern and Variable-Oriented Approach

**DOI:** 10.3389/fpsyg.2021.659300

**Published:** 2021-07-21

**Authors:** Laura Ferrer-Wreder, Kyle Eichas, Delia Stefenel, Carmen Buzea, Nora Wiium

**Affiliations:** ^1^Department of Psychology, Stockholm University, Stockholm, Sweden; ^2^Department of Psychological Sciences, Tarleton State University, Stephenville, TX, United States; ^3^Department of Social and Human Sciences, Lucian Blaga University of Sibiu, Sibiu, Romania; ^4^Department of Social Sciences and Communication, Transylvania University of Brasov, Brasov, Romania; ^5^Department of Psychosocial Science, University of Bergen, Bergen, Norway

**Keywords:** emerging adulthood, positive youth development, five Cs, latent class analysis, Romania

## Abstract

Transition to adulthood in countries that have seen recent historical structural changes in society as well as changes in what it means to be an adult represents important contexts for investigations of ways in which positive development and transition to adulthood are experienced. Situated in such context, this cross-sectional study aimed to (1) describe profiles of positive psychological strengths, as measured by the Five Cs of positive youth development (PYD) and (2) document how identified profiles might differ in relation to other aspects of positive and problematic development. Participants were 272 Romanian emerging adults attending university (76% female; aged 19–29 years old, *M*_age_ = 21 years old). Latent class analysis was performed to identify patterns of psychological strengths using the Five Cs theory of positive youth development. Pairwise Wald chi square difference tests were then conducted to determine if the identified Five Cs profiles were associated with significant differences in other key outcomes. Findings indicated that, a two-class model emerged as the best fitting model, and in this model, Class 1 was similar to Class 2 on strengths of competence, confidence, and connection. However, the two classes were distinguishable by caring and character, with Class 2, the numerically more common profile (89%), being elevated on character and caring relative to Class 1 (which was a less frequently occurring profile, 11%). This finding highlights the importance of examining the diversity of positive development, even within the same theoretical framework. Further results indicated that the two identified classes showed similarities in problematic behaviors as well as in purpose in life and psychological complaints. Other group difference tests by profile/class indicated that Class 2 was higher in general and social well-being relative to Class 1, with a trend in the same direction for hope. However, an unexpected finding was that Class 2 was also elevated in somatic complaints relative to Class 1. These are important findings not only because of the contribution to the generalizability of the Five Cs theory and measure but also because of the implications of the findings to research, policy, and practice in the Romanian context and beyond.

## Introduction

### Metatheoretical and Theoretical Foundations and Contributions

Within developmental science, there are many complementary metatheories of human development, with several views agreeing that a developing person and environment or context are complex, co-influential, and active over time (relational developmental systems or RDS metatheory—Overton, 2015; holistic-interactionistic view HI—Magnusson, 2001). A holistic view of the person was adopted in this study (consistent with elements of HI, Magnusson, 2001), and a pattern-oriented method was adopted in order to identify profiles of positive psychological strengths that in turn fell under the theoretical umbrella of the Five Cs model of positive youth development (PYD). An additional aim of this study was to examine if the identified profiles differ in other positive and problematic outcomes.

The first study aim with its pattern-oriented focus is needed, given that present day research on positive development is often characterized by studies in which a single construct or relations between a set of constructs/variables are the center of attention (Tolan et al., 2016). Variable-oriented (VO) approaches offer a view of what positive development could consist of across particular large groups of individuals. Yet, overreliance on one type of knowledge (e.g., nomothetic knowledge gained from VO approaches) is at odds with contemporary theories and metatheories about human development (e.g., RDS or HI), and maybe at odds with the nature of human development itself (Bergman and Andersson, 2010). As noted by Lerner et al. (2019, p. 10) “…development is about intraindividual change. Accordingly, these analyses *[variable-oriented]*^1^ do not illuminate the potentially unique individual pathways through which the PYD process unfolds…”

Several positive developmental theories, such as the Five Cs model of PYD, posit that individuals will make use of assets within contexts that fit their individual strengths and needs, and the nature of these developmental regulations between persons and contexts can change over time and place (Lerner et al., 2019). The person-oriented (PO) approach is a practical extension of HI metatheory that bridges HI concepts to everyday research practice. PO is not specific to the study on positive development but to human development in general (Bergman and Andersson, 2010) and seeks a closer correspondence between research practice and theory about human development. In PO “…the individual is regarded as a dynamic system of interwoven components that is best understood in terms of whole-system properties and often best studied by methods that retain these properties as far as possible, such as those that focus on individual patterns of information” (Bergman and Andersson, 2010, p. 155).

To address the first study research aim, a PO approach was used (*via* a type of pattern-oriented analysis) to examine psychological strengths of subgroups of individuals, with the expectation that there would be meaningful diversity in how the Five Cs of PYD patterned themselves within a group of individuals, particularly in context where little past similar research has been conducted. Further, this study added methodological novelty by combining PO and VO approaches in the second research aim to explore how the identified patterns of psychological strengths would be connected to other positive and problematic outcomes.

If positive development is not simply the avoidance or absence of problems and risk, then what does it consist of? According to Lerner, Lerner, and colleagues (Lerner et al., 2019), the Five Cs of PYD are important to understanding what it means for youth to thrive. According to Arbeit et al. (2014), within the Five Cs model, competence concerns a positive evaluation of one's ability to act in an effective manner in academic, social, and physical realms; confidence involves favorable self-worth as well as a sense of integrity about one's identity and body image; character consists of morality and values that have a sociocultural and personal basis, such as acceptance of diversity and responsibility; caring involves empathy toward others; and connection consists of the experience of mutually beneficial social bonds. It is further posited that when the Five Cs are prevalent part of the range of capabilities and dispositions of a young person, this will then lead to increased contribution to one's own development, to the development of others, and one's community (i.e., the Five Cs plus one; Lerner et al., 2019).

The Five Cs model was formulated in the early 1990s and was in part inspired by ideas prevalent in community-based youth development work in the United States (U.S.; Lerner et al., 2019). The process elements of this model are consistent with RDS metatheory (Overton, 2015) as well as other principles of human development, such as plasticity (i.e., the ability to change across development). RDS metatheory supports an emphasis on explaining growth in the Five Cs as a function of person context relations (Lerner et al., 2019). Within the model, there appears to be a theoretical openness to empirically testing and examining how the individual Five Cs should relate to one another in the widest possible variety of life conditions that are experienced by young people throughout the world (Lerner et al., 2019). Thus, the first research aim of this study is timely and needed, given that this study represents the first published examination of how the Five Cs of PYD pattern themselves in a sample of Romanian emerging adults.

As noted by O'Connor et al. (2016), scholars of positive development have pointed out the shortcomings of a one-sided view of young people as inherently deficit-rich and problematic. Yet, it is also the case that scholars who take a singular focus on only describing and explaining positive development offer an incomplete picture of youth and their development (O'Connor et al., 2016). A holistic view of the person (consistent with HI, Magnusson, 2001) provides a rationale for a wide-ranging focus that concerns how individuals function and adapt across domains of development (i.e., those that portend positive and problematic consequences in individual lives across time). In keeping with a holistic ethos, the second research aim of this study was to illuminate the potentially complex co-occurrence of aspects of positive (i.e., strengths such as those posited in the Five Cs model of PYD) and problematic facets of development or behavior. For example, one would expect a significant inverse association between, the Five Cs and antisocial behavior and alcohol use at the group or aggregate level. The results of the 4H study can provide some insights. This study was conducted by academics in partnership with a grassroots community-based national United States youth service organization called 4H. The longitudinal 4H study on PYD that included measurement of the Five Cs provided some evidence for this association at the group level, and yet other evidence at the subgroup level indicated some qualifications to this general association (Arbeit et al., 2014). For example, in a person-centered analysis of the 4H study, a subgroup of youth experiencing difficulties with externalizing behavior problems concurrently evidenced elevated levels of confidence and competence (Arbeit et al., 2014).

Thus, one can have few alternative hypotheses about the associations between strengths, such as the Five Cs of PYD and indicators of problematic development or behavior. One hypothesis could be a strong inverse association conceptualized as opposing poles of the same construct, with the positive (i.e., strengths) on one side of a continuum and the problematic on the other side (e.g., cumulative risk models imply this association; O'Connor et al., 2016). Other hypotheses could be that strengths and problems are not related to each other or that strengths and problems are associated with each other but have a moderate association because this association may only be the case for a particular subgroup and not others, and this variation by subgroup dampens the overall association at a group level (O'Connor et al., 2016).

These alternative hypotheses have vital implications for intervention efforts, in that if there is a strong inverse association between strengths and problems, basically opposing poles of the same construct, interventions designed to boost strengths will also address problems (O'Connor et al., 2016), or, if there are complex relations and co-occurrences at work, then simultaneous and coordinated implementation of promotion, prevention, and treatment approaches tailored to subgroups maybe warranted and ultimately of greatest benefit (O'Connor et al., 2016). Thus, this study begins to address these issues in a Romanian context for individuals who are in transition to adulthood.

The other broad conceptualization important to this study is the specificity principle within the field of human development (Bornstein, 2017). Developmental science has been characterized by a search for universal features and processes that explain human development on a global scale. Applied to positive development, this would imply that a certain combination of facets of positive development is indeed adaptive and is at the foundation of thriving universally across the wide range of life experiences and conditions that young people experience during the transition into adulthood across the world and across historical time. Bornstein (2017) argued that universals in human development can co-exist with the observation that a wide diversity of life experiences and conditions can produce diversity in development. Applied to positive development, this would mean that there could be commonalities across the globe in some valued and adaptive facets of positive development, but other facets may be of value only under some conditions and/or points in history because of prevailing socio-cultural and historical conditions (e.g., community engagement and contribution). Further, as noted by Lerner et al. (2019, p. 10) “…the constructs pertinent to any PYD model may be manifested differently in different national or cultural contexts. Clearly, future research needs to address these possibilities.” Thus, the contribution of this study is clear in that it is one of only a handful of studies about the positive development of Romanian emerging adults attending university, and no study published to date has used a pattern-oriented approach.

### Socio-Cultural and Historical Context of This Study

In keeping with the specificity principle (Bornstein, 2017), it is important to document the sociocultural and historical context in which this study was conducted. Culturally speaking, Romania is a non-Western, educated, industrialized, rich and democratic (WEIRD) country (Henrich et al., 2010). The mainstream culture of Romania has been characterized as collectivistic (i.e., loyalty to family or to other significant groups as central societal norm), with high degree of power distance (i.e., hierarchies exist and are accepted to some degree) and elevated avoidance of uncertainty (i.e., rigid codes of belief and behavior, with low tolerance for novelty and unusual conduct) (Hofstede, 2020). The Romanian culture has also been described as scoring low on indulgence, in terms of people likely to undervalue their own personal desires and restrain their action to keep in step with social norms (Hofstede, 2020). However, like other East-Central European countries, Romania scores high on intellectual autonomy, understood in terms of creativity, curiosity, freedom, broad-mindedness, and harmony as well as low on hierarchy (Schwartz, 2008).

In a comprehensive monograph about the psychology of Romanians, David (2015) presented a detailed analysis of the global national psychological/personality profile of Romania. It was found that Romanians have average levels of neuroticism (i.e., anxiety, hostility, depression, self-consciousness/shyness, impulsiveness, and vulnerability to stress) and average levels of agreeableness (i.e., trust, straightforwardness/honesty, altruism, compliance, modesty, and tenderness). David (2015) also showed that Romanians have high levels of extraversion (i.e., warmth, gregariousness, assertiveness, activity, excitement-seeking, and positive emotion), openness (i.e., willingness for new experiences in terms of aesthetics, feelings, actions, ideas, and values), and conscientiousness (i.e., competence, order, dutifulness/honor, achievement striving, self-discipline, and deliberation).

Economically speaking, Romania is a post-communist Eastern European country with an upper-middle national economy. After the fall of the Iron Curtain, the country provided a completely new and challenging environment for both adults and young people, given the transition from communism to democracy (Diamond, 2015), with a free-market economy and access to the European Union (EU). Given its cultural and historical context, Romania represents an important context for studying the transition to adulthood, given that education and positive youth development initiatives are still underdeveloped within Romania. For example, Romanian investment in education remains low in comparison with other EU countries (European Commission, 2019). Furthermore, tertiary educational attainment of 24.6% for Romanian youth is far below the EU average of 40.7%, while early school leaving of youth aged 18–24 years old is one of the highest in the EU (16.4 compared with 10.6% EU average; European Commission, 2019). In Romania, youths make the transition to adulthood in an environment affected by poverty and structural inequality (Bădescu and Sum, 2015), as well as fear of corruption and social injustice (Bădescu et al., 2019). The transition to adulthood in Romania for young people is also marked by lack of employment opportunities after graduation from school, with approximately 17% of young people being not employed or in further education or training (i.e., NEET, Eurostat, 2019).

With less than optional educational and economic supports for successful transition into adulthood, the emotional life, well-being, and health of Romanian children, adolescents, and emerging adults are also important to reflect on as part of the context for this study. Health Behavior in School-aged Children (HBSC; World Health Organization, 2020) responses from Romania indicated that approximately one in four children report somatic and psychological problems, such as difficulty in falling asleep and unhappiness, at least once a week. Youths in Romania report higher than average alcohol use (relative to European averages; World Health Organization, 2018) and elevated rates of experiences with bullying (World Health Organization, 2020). In terms of social adjustment problems, such as substance use, in a study with 1,359 Romanian young adults (18–30 year-olds), only 3% of participants were abstinent, with main reasons for alcohol use being reported as: curiosity (67.8%), to be liked by peers (17.9%), adult influence (6.5%), and boredom (4.8%; Rada and Ispas, 2016). The national drug report for 2019 also noted that the prevalence in use of illicit substances among those aged 15–34 years old increased; in the case of cannabis for example, the number of users doubled from 2013 to 2016 (European Monitoring Center for Drugs and Drug Addiction, 2019). Furthermore, within Romania there is concern about the loss and outflow of human capital out of the nation *via* immigration of young people out of Romania (Romanian National Statistics Institute, 2019). Romanian youth and emerging adults represent a clear resource to their society and its future. Documenting the unique configuration of strengths that Romanian emerging adults possess is vital and maybe important to understanding adjustment and thriving of emerging adults in other European post-communist nations that are facing similar social and historical challenges.

### Prior Studies on the Positive Development of Romanian Youth and Emerging Adults

Prior research studies that can inform this study are few in numbers and mainly come from studies in the international research literature outside of Romania. There are a handful of prior research studies that address and describe the emerging adulthood experience, in general, in contemporary Romania. For example, Vincze et al. (2013) found that the vast majority (e.g., 78–94%) of 18- to 25-year-old Romanian respondents in their study reported that they saw maturity as being characterized by the experience of freedom, responsibility for the actions that one takes, independent decision-making, and financial independence.

Within the realm of positive development, there are also some VO studies that have begun to document facets of positive development (e.g., Karaś et al., 2014). For example, Negru (2012) found variation in which factors predicted greater life satisfaction among Romanian high-school and university students. For example, high school students were likely to be more satisfied with life if they were low on identity exploration, and had more self-focus and increased feelings of being in-between; while for university students, increased satisfaction with life was predicted by high experimentation/perception of possibilities, high self and other focus, and low negativity/instability. Dimitrova et al. (2017) investigated ethnic and national identity resources underlying positive adaptation in Roma minority youth in several countries, such as Romania (i.e., Albania, Bulgaria, the Czech Republic, Kosovo, Italy, and Romania). Results indicated that among Roma samples across nations, the ethnic identity of Romanian Roma youth was lowest, and that for the Romanian Roma youth their ethnic identity was not significantly associated with self-esteem, yet elevated national identity (an identity rooted in the mainstream Romanian culture) was positively related to self-esteem.

Although the content focus of these studies (i.e., Negru, 2012; Dimitrova et al., 2017) differs from this study, an important lesson learned from these previous investigations is that subgroups of Romanian youth and emerging adults may differ from one another in important ways in the observed connections between facets of positive development and adjustment or problems. The subgroups may be defined by whether the young person is an adolescent or emerging adult, or it may be defined by one's position and relation to the mainstream culture, whether youth are in the Romanian cultural mainstream or part of an ethnic minority group, such as having a Roma heritage. Subgroups based on developmental period or ethnicity are not examined in this study, but the focus is, instead, on subgroups, as defined by empirically identified profiles of positive psychological strengths (i.e., the Five Cs), and how these subgroups may differ from one another in regard to other strengths and problems.

### The Study

Since this study was exploratory and there was little direct relevant research to base specific hypotheses on, this study was guided by research questions that have a theoretical basis, not *a priori* hypotheses. In keeping with the PYD theory (Lerner et al., 2019), we examined if there was meaningful diversity in how the Five Cs of PYD patterned themselves in this sample. This is also consistent with a person-oriented focus on the whole system and its properties, in this case the Five Cs of PYD model. However, there were no hypotheses about a specific number of Five Cs patterns to identify or how identified patterns would relate to differences in problematic outcomes (i.e., somatic and psychological complaints as well as substance use and antisocial behavior). Thus, in this study, there was openness to any of the hypotheses about strengths and problems that were put forward by O'Connor et al. (2016), namely, strong inverse association, strengths and problems unrelated, or a moderate association depending on subgroup.

The positive outcomes examined in this study, purpose, hope, and well-being, have theoretical roots within positive psychology (Seligman and Csikszentmihalyi, 2000). Positive psychology and the Five Cs of PYD are distinct but allied subfields within the study of positive development (Tolan et al., 2016). In advocating for a more integrative approach to positive developmental research, Tolan et al. (2016) noted that points of possible conceptual alignment between key constructs in the Five Cs and positive psychology include a common meta construct of *Social Bonds and Morality*, as indexed by caring in the Five Cs and meaning (i.e., purpose) in positive psychology. Also, according to Tolan et al. (2016), the meta-construct of *Positive Self-Orientation* could be indexed by positive emotions, such as well-being and hope (examined within positive psychology), and confidence in the Five Cs model. The array of positive outcomes included in this study (the Five Cs along with constructs from positive psychology) contributes to efforts that advance the study of positive development by conducting research in which constructs from more than one theoretical perspective/model are included and thereby has the potential to shed light on instances when models empirically overlap and when unique value is added by a particular model. Thus, how any identified patterns of the Five Cs would relate to differences in specific positive outcomes (i.e., hope, purpose, and well-being) was also not posited because of uncertainty regarding what the identified patterns would be characterized by.

### Study Aims and Research Questions

This study has two aims. The first aim was to describe profiles of positive psychological strengths, as measured by the Five Cs of PYD. This aim was addressed by research question 1 (RQ1): Are there distinct patterns of the Five Cs of PYD that characterize subgroups of participants within this sample?

The second study aim was to document the ways in which identified profiles may differ in relation to other aspects of positive and problematic development. This aim relates to research question 2 (RQ2): Do the identified patterns of the Five Cs evidence mean level differences in positive or problematic outcomes? RQ2 allows for an exploration of the concept of conceptual alignment between positive psychology and the Five Cs model of PYD, as well as exploration of the possible associations between strengths and problems as outlined by O'Connor et al. (2016).

## Materials and Methods

### Participants

The participants were students at a public university in Central Romania (*N* = 272; 76% female; aged 19–29 years old, *M*_age_ = 21 years old) and were mainly students in the Social and Human Sciences. They were primarily pursuing bachelor's degrees (92%) with few taking up master's degree studies (8%). Most of the participants (64%) lived with one or both their parents, few (23%) lived alone, and approximately 13% had other living arrangements (e.g., living with an adult who was not a parent). They described their homes as being located in a large town or city (50%), small town (28%), or village (22%). The most frequently reported highest education for parents of the participants was: university or college (19% fathers; 25% mothers), technical or vocational school (73% fathers; 68% mothers), or secondary school (2% fathers; 4% mothers). With regard to religion, the majority of the sample (85%) reported that they were Orthodox.

### Measures

The factor structure and latent reliability of the instruments used in this study are described in greater detail subsequently in the results section of this article and in Table 1 as well as Appendix 1 in Supplementary Material. This section is a description of the instruments themselves as administered to this sample and a summary of the measurement analyses described in the results and tables. Scales in this study are well used in the international research literature on adolescent and/or emerging adult development and health, and have evidenced generally acceptable to very good psychometric properties (e.g., van Gestel-Timmermans et al., 2010; Geldhof et al., 2014a; Hill et al., 2016).

**Table 1:** Factor loadings and measurement model.

**Factor**	**Items**	**Standardized estimate (95% CI)**
Competence	Comp_p1	0.695 [0.586, 0.803]
	Comp_p2	0.687 [0.588, 0.787]
Confidence	Conf_p1	0.851 [0.793, 0.909]
	Conf_p2	0.911 [0.869, 0.953]
Character	Char_p1	0.448 [0.322, 0.574]
	Char_p2	0.804 [0.728, 0.880]
	Char_p3	0.849 [0.779, 0.920]
Caring	Care_p1	0.866 [0.813, 0.633]
	Care_p2	0.801 [0.726, 0.875]
	Care_p3	0.887 [0.840, 0.933]
Connection	Conn_p1	0.848 [0.773, 0.923]
	Conn_p2	0.813 [0.749, 0.877]
	Conn_p3	0.570 [0.466, 0.675]
Wellbeing	WB_ewb	0.747 [0.671, 0.824]
	WB_swb	0.670 [0.600, 0.741]
	WB_pwb	0.865 [0.817, 0.913]
Hope	Hope_p1	0.817 [0.753, 0.881]
	Hope_p2	0.873 [0.828, 0.918]
	Hope_12	0.717 [0.634, 0.800]
Purpose	Purp_p1	0.815 [0.752, 0.878]
	Purp_p2	0.935 [0.879, 0.991]
Somatic complaints	Soma_p1	0.800 [0.705, 0.895]
	Soma_p2	0.587 [0.482, 0.692]
Psychological complaints	Psyc_p1	0.762 [0.676, 0.848]
	Psyc_p2	0.776 [0.703, 0.850]
Substance abuse	SA_score	0.922 [0.906, 0.939]
Antisocial behavior	AB_score	0.863 [0.810, 0.915]

#### Purpose in Life Short Form

The Purpose in Life Short Horm (Hill et al., 2016) consists of four items that concern one's direction in life as well as the alignment of one's direction in life with authentically held views, interests, and commitments. A sample item is “There is a direction in my life.” Items are rated on a 5-point Likert scale from *Strongly Disagree* = *1* to *Strongly Agree* = *5*. See Appendix 1 in Supplementary Material for results from confirmatory factor analysis (CFA) and item examples. The composite reliability estimate for the retained one factor latent model with four items was 0.93 (McDonald's ω).

#### Herth Hope Index

The Herth Hope Index consists of 13 items (Herth, 1991; van Gestel-Timmermans et al., 2010). Hope is operationalized in this instrument as consisting of having a positive view on life and one's future as well as having a sense of meaning in life and beneficial connections to others. A sample item is “I have a positive outlook toward life.” Items have 11 positively and two negatively worded items that are rated on a 5-point Likert scale from *Strongly Disagree* = *1* to *Strongly Agree* = *5*. See Appendix 1 in Supplementary Material for CFA results and item examples. The composite reliability estimate for the retained one factor latent model with five items was 0.87 (McDonald's ω).

#### Subjective Health Complaints

A scale from the Health Behavior in School-aged Children (HBSC) survey (Inchley et al., 2018; Cosma et al., 2020) was used as an index of subjective health complaints. The participants reported on the recent (past 6 months) frequency of eight somatic and psychological symptoms, which were, for example, experiencing “*headache*,” “*stomach ache*” (four somatic items), and for psychological symptoms, for example, “*feeling low/depressed*,” “*feeling nervous*” (four psychological items). Response options are rated on a 5-point Likert scale *from Rarely or Never* = *1 to About Everyday* = *5*, with higher scores indicating a greater frequency in symptoms. See Appendix 1 in Supplementary Material for CFA results and item examples. The composite reliability estimate for the retained two-factor latent model with four items on each factor was 0.75 (McDonald's ω) for the somatic factor and 0.89 (McDonald's ω) for the psychological factor.

#### Mental Health Continuum Short Form

The Mental Health Continuum Short Form (Keyes et al., 2020) consists of 14 items that concern well-being with regard to emotional states (e.g., “happy”) as well as aspects of well-being in general (e.g., “…life has a sense of direction or meaning to it”), social well-being (e.g., “…belonged to a community…”), and psychological well-being (“…like most parts of your personality”). The frequency of experiencing these aspects of well-being is rated on a 6-point Likert scale from *Never* = *1 to Everyday* = *6*, with higher scores indicating greater well-being/thriving. Items on this scale can be used in a categorical (cut offs are used to determine thriving on a certain number of items rating oneself as a five or six) or continuous manner. See Appendix 1 in Supplementary Material for CFA results and item examples. The composite reliability estimate for the retained modified bi-factor latent model with 14 items was 0.93 (McDonald's ω) for the general factor, 0.8 (McDonald's ω) for the emotional factor, 0.8 (McDonald's ω) for the social factor, and 0.68 (McDonald's ω) for the psychological factor.

#### Short Form Five Cs of PYD Measure (PYDSF)

The Five Cs of PYD (competence, confidence, character, caring, and connection) Short Form consists of 34 items (PYDSF; Geldhof et al., 2014a) rated on a 5-point Likert scale. However, the response options on the 5-point scale can vary depending on the item/scale. Items can be averaged into one of five theory-based scale scores (Geldhof et al., 2014a). On the short form, appraisal of competence of the participants in social relations, academic life, and athletics (e.g., “*I am just as smart as others my age*”) can be measured with six items rated from *Strongly Disagree* = *1* to *Strongly Agree* = *5*. View of the confidence that the participants have in themselves can be measured by six items. These items concern satisfaction with oneself in general (i.e., self-worth and positive identity) or satisfaction with one's physical appearance. Items on this scale are rated *Strongly Disagree* = *1* to *Strongly Agree* = *5*. Character can be measured with eight items that concern behavioral conformity to norms (e.g., sample item: “*I usually act the way I am supposed to*” rated *Strongly Disagree* = *1* to *Strongly Agree* = *5*), as well as items on the acceptance of prosocial norms or social conscience. Items in this case are rated as *Not Important* = *1 to Extremely Important* = *5*. Other items on this scale concern endorsement of values about cultural diversity, with these items rated as *Not at All Like Me* = *1 to Very Much Like Me* = *5*. Caring can be indexed by six items that concern empathy toward others in terms of affect and behavior with these items rated as *Not at All Like Me* = *1 to Very Much Like Me* = *5*. Connection is measured by eight items that regard perception of belonging and acceptance of the participants from important others at university, in one's community, as well as among family and friends. Two items about friends are rated *1* = *Almost Never True or Never True to 5* = *Always True* and the remaining items are rated *Strongly Disagree* = *1* to *Strongly Agree* = *5*. See Appendix 1 in Supplementary Material for CFA results and item examples. The composite reliability estimate for the retained modified five-factor latent model with 28 items was 0.63 (McDonald's ω) for competence (four items), 0.91 (McDonald's ω) for confidence (four items), 0.71 (McDonald's ω) for character (six items), 0.92 (McDonald's ω) for caring (six items), and 0.71 (McDonald's ω) for connection (eight items).

#### Substance Use

Seven items from the Search Institute Attitudes and Behaviors: Profiles of Student Life (Leffert et al., 1998) were used to index recent (last 30 days) alcohol and tobacco use (three items for example “Have you used alcohol once or more in the past 30 days”) as well as use of illicit drugs or being involved with drunk driving (as a driver or passenger) in the past 12 months (four items). These seven items were rated with a yes or no response. See Appendix 1 in Supplementary Material for CFA results and item examples. The composite reliability estimate for the retained one-factor latent model with seven items was 0.88 (McDonald's ω).

#### Antisocial Behavior

Six items from the Search Institute Attitudes and Behaviors: Profiles of Student Life (Leffert et al., 1998) were designed to index several different antisocial behaviors in the past 12 months, ranging from gambling and skipping classes to shoplifting, vandalism, carrying a weapon, and getting involved in a group fight. These items were rated as yes or no. See Appendix 1 in Supplementary Material for CFA results and item examples for this scale. The composite reliability estimate for the retained modified one-factor latent model with five items was 0.78 (McDonald's ω).

### Procedure

This cross-sectional study was conducted as part of a wider cross-national study on positive development (Wiium and Dimitrova, 2019). The study used self-report (paper and pencil) surveys administered in a group classroom setting with university students in Social Sciences and Humanities. Scales and items not available in Romanian from previous studies were translated using a committee approach that involved a team of Romanian academic staff and students collaborating in an iterative process of translation until consensus was reached (van de Vijver, 2019). The team members translated the instrument independently, and they met in two rounds: one for review and refinements and one for mediation and deciding on revised version. The paper-and-pencil survey administration typically took no more than 30 min and was anonymous. Informed consent was obtained prior to data collection, and no participants declined to take part in the study. Study location was a public university in Central Romania. The participants received no incentives or credits for study participation. As part of the cross-national study on positive development, this study received ethical approval from the NSD-Norwegian Center for Research Data.

### Data Analysis Strategy

Data analysis was undertaken in three main steps. First, we evaluated the factor structure of each measure individually with CFA. If necessary, modifications were made to the hypothesized measurement models based on the CFA analyses and guided by theory and prior psychometric studies. Second, we examined the factor correlations among all the constructs by including them in a single CFA model. The size of the model was reduced through the use of a parceling strategy, as discussed below. Third, we conducted an unconditional latent profile analysis of the PYDSF subscale scores for the Five Cs of PYD (competence, confidence, character, caring, and connection) and then compared the class means on the other variables. All analyses were conducted using Mplus 8.4 for structural equation modeling. For tests of absolute model fit, we examined the chi square test of exact fit and its *p*-value, comparative fit index (CFI), Tucker–Lewis index (TLI), root mean square error of approximation (RMSEA), and standardized root mean square residual (SRMR). Good model fit was indicated by: chi square *p* < 0.05, CFI > 0.95, TLI > 0.95, RMSEA < 0.06, and SRMR < 0.08 (Hu and Bentler, 1999).

## Results

### Measurement Model

First, we constructed a measurement model for the latent variables. Indicators of most of the latent variables were parcels constructed (i.e., a procedure normally used in structural equation modeling analysis to compute sums or average scores across multiple items to improve the quality of indicators and model fit while reducing model complexity) based on factor loadings from the item-level factor analyses. The exceptions were General Well-being, Substance Use/Abuse, and Antisocial Behavior. General Well-being was measured with parcels for Emotional Well-being, Social Well-being, and Psychological Well-being to reflect the bi-factor structure of Well-being. Specifically, we computed an average Emotional Well-being score, average Social Well-being score, and average Psychological Well-being score and modeled these three scores as indicators of General Well-being. Substance Use/Abuse and Antisocial Behavior were both modeled as single indicators, because the scales used to measure these constructs comprised binary items. Instead, the single indicators were modeled with corrections for measurement error that corresponded to reliability estimates of 0.85 for Substance Use/Abuse and 0.75 for Antisocial Behavior.

All other parcels were created using a balancing approach in which the strongest loading item on a factor was parceled with the weakest loading item, the second strongest with the second weakest, and so forth (Little et al., 2013). Scores on the items within each parcel were averaged to create indicators of the outcome variables. We estimated this model with a robust maximum likelihood estimator. The test of exact model fit was rejected based on a statistically significant chi square value, χ^2^(260) = 419.079, *p* < 0.001. Most fit indices were consistent with acceptable fit [CFI = 0.95, RMSEA = 0.05 (0.04, 0.06), SRMR = 0.05]. The exception to this was the TLI, which was slightly below the cut-off, 0.94. We retained this model. Table 1 provides the factor loadings for the measurement model. Details about steps involved in the development of these measurement models are shown in Supplementary Material, and Appendixes 1 and 2.

### Latent Profile Analyses and Group Difference Tests

Next, we conducted a series of analyses to test the main study aims. The first aim concerned describing how the Five Cs of PYD patterned themselves in this sample. The analyses performed to examine this aim were latent profile analysis of the PYDSF subscale scores for the Five Cs of PYD (competence, confidence, character, caring, and connection). We tested and compared two-class, three-class, and four-class models using the three-step process suggested by Asparouhov and Muthén (2012). Consistent with the exploratory nature of latent profile analysis, the number of classes we tested was guided by model fit information, as described below. Each model included residual covariances among the Five Cs and was estimated with a robust maximum likelihood estimator. First, we obtained the best loglikelihood values for the two-, three-, and four-class models. In a first run for each model, we requested 200 sets of start values in the first optimization step and 40 sets of starts in the second optimization step. In a second run, we increased the number of requested sets of start values to 600 and 120, respectively, to ensure that the best loglikelihood value was replicated. Second, we used the sets associated with the best loglikelihood value in the first step to compare each model with a more parsimonious model with one fewer class by the Vuong–Lo–Mendell–Rubin (VLMR) test. Third, we again used the sets associated with the best loglikelihood value in the first step to compare each model with a more parsimonious model with one fewer class, this time by the bootstrap likelihood ratio test (BLRT). In a first run for each model, we used the Mplus default of 20 and 5 sets of start values for the first and second optimization steps for the more parsimonious model. If the best loglikelihood value was not replicated, then we increased the number of requested sets of start values to 100 and 20 in a second run and, if necessary, to 500 and 80 in a third run.

The results from the model comparisons are shown in Table 2. Lower BIC values indicate better relative fit, while statistically significant VLMR and BLRT *p*-values indicate that a model fits better than a more parsimonious model with one class less. All three indices suggested that the two-class model fit better than the one-class model. VLMR suggested that the three-class model did not fit better than the two-class model, whereas BLRT suggested the opposite. Similarly, VLMR suggested that the four-class model did not fit better than the three-class model, but BLRT suggested the opposite. Of all the three models, BIC supported the two-class model. Since VLMR and BIC indicated that more than two classes did not improve model fit, we did not explore more complex models with five or more classes. Instead, we selected the two-class model for further analysis.

**Table 2:** Model comparisons and latent profile analysis of the Five Cs.

**Model**	**Loglikelihood**	**BIC**	**Entropy**	**VLMR**	***p***	**BLRT**	***p***
2 classes	−1178.965	2503.680	0.896	50.137	<0.001	50.137	<0.001
3 classes	−1165.111	2509.608	0.815	27.702	0.342	27.707	0.013
4 classes	−1154.363	2521.746	0.851	21.496	0.382	21.496	<0.001

**Figure 1:**
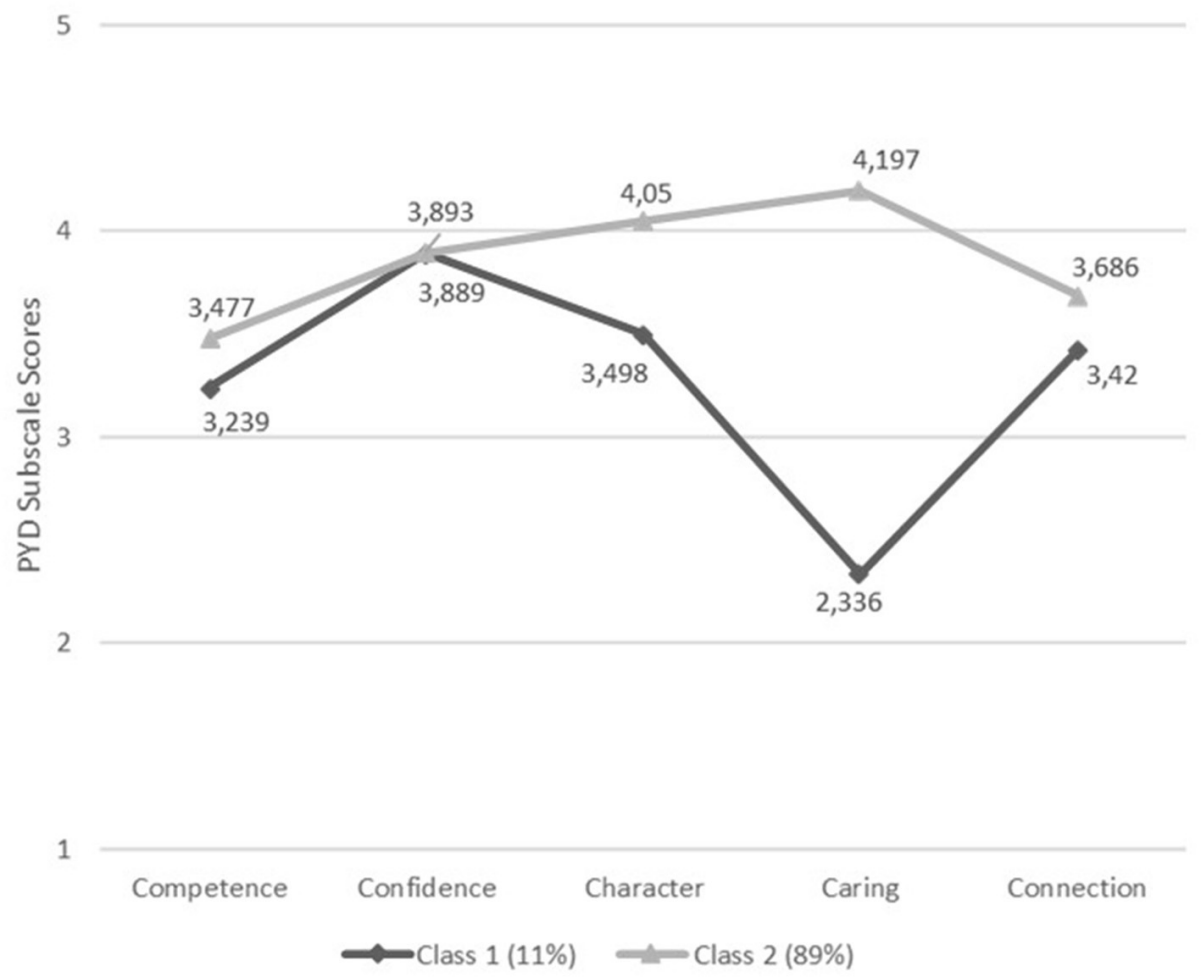
A profile plot for the 2-class model.

The profile plot for the two-class model is shown in Figure 1. Class 1, which included 11% of the cases, had means that were similar to those of Class 2 on competence, confidence, and connection. However, the Class 1 mean for caring was 1.861 points lower than the Class 2 mean for caring. The Class 1 mean for character was 0.552 points lower than the Class 2 mean for character.

The second study aim explored how the identified profiles might differ in other aspects of positive and problematic development. This was done by using the 3-step approach described in Nylund-Gibson and Choi (2018) for examining distal outcomes in latent class analysis. Specifically, we compared the two identified classes on the other outcome variables (purpose in life, hope, somatic complaints, psychological complaints, well-being, substance use/abuse, and antisocial behavior) by Wald $\chi_{\text{diff}}^{2}$ tests. Results are shown in Table 3.

**Table 3:** Difference tests, Class 1 vs. Class 2.

			**Class means**		
**Variables**	**Wald** $\chi_{\text{diff}}^{2}$**(df)**	***p***	**Class 1**	**Class 2**	**Difference**
Purpose in life	0.702(1)	0.402	3.654	3.809	−0.156 [−0.520, 0.208]
Hope	3.469(1)	0.063	3.633	4.078	−0.446 [−0.915, 0.023]
Somatic complaints	3.852(1)	0.050	1.733	2.084	−0.352 [−0.703, 0.000]
Psychological complaints	0.942(1)	0.332	2.600	2.794	−0.194 [−0.587, 0.198]
General well-being	3.891(1)	0.049	3.683	4.117	−0.434 [−0.864, −0.003]
Emotional well-being	0.970(1)	0.325	4.280	4.624	−0.344 [−1.028, 0.340]
Social well-being	4.834(1)	0.028	2.897	3.394	−0.497 [−0.940, −0.054]
Psychological well-being	1.292(1)	0.238	3.957	4.294	−0.338 [−0.898, 0.223]
Substance use/abuse	0.247(1)	0.619	1.755	1.910	−0.155 [−0.769, 0.458]
Antisocial behavior	–	–	0.000	0.428	−0.428 [–,–]

Among the four negatively oriented outcomes, which included somatic complaints, psychological complaints, substance use, and antisocial behavior, only somatic complaints showed a significant average difference between the Classes. Specifically, Class 1 evidenced a lower average score than Class 2 on somatic complaints. Class 2 was the more commonly occurring profile (89% of cases) that was higher (than Class 1) on the 5Cs caring and character scales. Thus, being elevated on caring and character was also connected with more somatic complaints. Otherwise, there were no significant differences between the identified Classes on the other negative outcomes examined in this study.

Among the six positively oriented outcomes (purpose, hope, general well-being, emotional well-being, social well-being, psychological well-being) the results showed more differences among the Classes. Class 2, in comparison with Class 1, was significantly elevated on general well-being and social well-being. In other words, the Class that was higher on the 5Cs caring and character (i.e., Class 2) also showed greater general and social well-being in comparison with Class 1 (11% of cases). There was also a trend toward a significant difference (*p* = 0.06) in the same direction for hope, with Class 2 approaching a higher score on hope relative to Class 1.

## Discussion

The two aims of this study were to describe the profiles of positive psychological strengths, as measured by the Five Cs of PYD, and to document the ways in which positive and problematic development can co-occur. For the first research aim, a two-class model emerged as the best fitting model compared with a one-, three-, and four-class model. Focusing on the two-class model, Class 1 was similar to Class 2 on the strengths of competence, confidence, and connection. However, the two Classes were distinguishable on caring and character, with Class 2, the numerically more common profile in this sample (89%) being elevated on character and caring relative to Class 1 (which was a less frequently occurring profile in this sample, 11%). This finding, while descriptive in nature, highlights the importance of examining the diversity of positive development, even within the same theoretical framework (the Five Cs theory) as well as documents how positive psychological strengths are not uniformly experienced across a group of individuals.

Further, in relation to the second research aim, the two identified Classes showed some similarities on their reports of problematic behaviors, such as antisocial behavior, substance use as well as on some positive aspects of development, such as purpose in life and on an index of health, namely psychological complaints. Other group difference tests by profile/class indicated that Class 2 was higher on general and social well-being relative to Class 1. A trend in the same direction was found for hope. However, an unexpected finding was that Class 2 was also elevated on somatic complaints relative to Class 1.

That two profiles of the Five Cs were found, one reflecting above average to high levels of the Five Cs and the other reflecting a profile of low to above-average levels, suggests that higher levels of the PYD indicators do not always cluster together. It should be noted that in the use of the term “average” in this instance, we are referring to “average” on a one to five scale (i.e., 3), which is the scale used on the Five Cs instrument. Normative data on the Five Cs for Romanian emerging adults is presently not available. Indeed, earlier studies have found these patterns in general population youth samples. For example, in a study among a sample of Irish adolescents, higher scores were found for caring and connection, while participants scored relatively low on competence (Conway et al., 2015). Among emerging adults in Ghana, who are similar to the participants in this study in terms of age and educational background, scores on caring and confidence were relatively high while those for competence were relatively low (Wiium et al., 2019). In this study, the score on competence is the lowest for both PYD profiles, while Class 1 has the highest score on confidence and Class 2 on caring. For Class 2, there is a clustering of the Five Cs at higher levels (although not entirely), indicating thriving, which, in line with PYD theoretical perspective and empirical evidence, should lead to more contribution to self, family, community, and society (Lerner et al., 2005).

With youth and emerging adults in Romania facing social, economic, and educational challenges that can translate into poverty, social adjustment problems, low tertiary educational attainment, high unemployment rate, and structural inequality, among others, identifying factors that facilitate thriving among emerging adults is critical. It is, therefore, encouraging that a PYD profile that reflects clustering of relatively high scores on the Five Cs was identified and associated with a class that contained majority (89%) of the 272 participants involved in this study. However, not all have healthy levels of the thriving indicators, suggesting that more work needs to be done to promote thriving among young Romanian people, an assertion, which is especially true for emerging adults, as they transition to full adult roles.

The findings also revealed associations between the two PYD profiles and several indicators of well-being. At the earlier stage of positive youth development research, positive youth outcomes, such as the Five Cs, were theorized to relate inversely to risk and problem behaviors (e.g., depression, violence, and substance abuse). This assertion has been confirmed in empirical research on associations between average PYD indicator scores and problem behaviors (Geldhofet al., 2014a). However, research that has examined the profiles of the Five Cs in longitudinal studies (using person-centered analysis rather than the often-used variable centered analysis) has indicated a more complex association. Accordingly, in longitudinal studies, while an inverse relation has been observed, the two outcomes have also been found to relate positively to each other in some trajectories, or not related at all in others (Lewin-Bizan et al., 2010).

In this study, Class 1was related to a lower average score on Somatic complaints relative to Class 2. This was an unexpected finding. One possible reason for Class 2 being associated with a higher score on somatic complaints could be due to their high score on caring. Although caring, like the other Cs of PYD, is perceived as a thriving indicator, earlier research studies among youths in Lithuania (Truskauskaite-Kunevičiene et al., 2014), Norway (Holsen et al., 2017) and the United States (Geldhofet al., 2014b) have also reported a positive link between the PYD indicator of caring and internalizing problems, such as depression and anxiety. Geldhof et al. (2014b) found this positive link after controlling for the global measure of PYD and argued that high levels of empathy and sympathy in youth, as reflected in caring, may cause them to be overconcerned about thoughts and feelings of other people. In this study, the two PYD profiles, which differed significantly on caring, did not differ on psychological complaints (measured as emotional and mental feelings of an individual). They differed, rather, on somatic complaints, which were measured by report of headaches, stomach aches, and backaches, among others, of the participants. However, somatic complaints tend to correlate positively with psychological complaints (Lyyra et al., 2018). While the above explanations may hold, it is also possible that the positive link between Class 2 and somatic complaints was due to other unmeasured factors, and this unexpected finding certainly warrants more attention in future studies.

The current findings also showed positive associations between Class 2 and several distal positive outcomes, such as hope, general well-being, and social well-being. These associations are not surprising, as they align with both the theoretical assumptions and empirical evidence of the PYD perspective. Theoretically, the Five Cs have been hypothesized to be related to a sixth C, contribution, to self, family, community, and society. Empirically, the effect of the Five Cs (either as a global factor or five different components) on contribution and other positive indicators has been observed (Lerner et al., 2005; Holsen et al., 2017). The current findings add to the empirical evidence in that the Five Cs reported among Romanian emerging adults attending university were also found to be related to several aspects of their well-being or positive indicators (hope, general well-being, and social well-being).

Returning to theoretical debate on how strengths and problems are associated with each other (O'Connor et al., 2016), the findings support the conceptualization that strengths and problems are distinct constructs, and that subgroup processes are at work, at least in this sample. As noted, this type of theoretical conceptualization implies for intervention work that simultaneous and coordinated promotion and prevention approaches tailored to subgroups may be ultimately of greatest benefit (O'Connor et al., 2016). The findings only allow for provisional conclusions and feedback into this theoretical debate and await replication or qualification by future research studies with diverse Romanian emerging adults (those in and outside of university).

### Limitations

Despite the novelty and importance of the study findings, there are also several limitations. First, although we had enough of a sample to be able to draw meaningful conclusions about the participants, this sample size was still relatively small and non-representative of the complete emerging adult population in Romania (which includes non-university emerging adults) and was predominately female. For context, the education profile nationally is that 9.9% of people aged 15–29 are enrolled in higher education programs (Eurostat, 2020), so many Romanian emerging adults are not represented by studies conducted at a university. Further, the sample size coupled with the uneven distribution of gender limited the ability to conduct subgroup analysis by gender. It is important to note that this sample was representative in terms of religious affiliation as predominately orthodox, which makes up the main religious affiliation in the total population (86.45%; NIS [National Institute of Statistics], 2013). The only other socio demographic indicator recorded in this study was highest education of parents, with participants reporting: university or college (19% fathers; 25% mothers), technical or vocational school (73% fathers; 68% mothers), or secondary school (2% fathers; 4% mothers).The highest education for parents of the participants in this study is consistent with the national education profile (Eurostat, 2020), in that Romanians aged 35–54 (the likely age of the parents of the participants in this study) have completed higher education at approximately similar levels (18% males, 21% females), as well as technical or vocational school (65% males, 60% females). Sociodemographic factors could have played a moderating role in the findings of this study. However, these factors were not tested in this study. It would be of benefit to have additional sociodemographic indicators collected (e.g., family income) with a larger sample size with emerging adults in and outside of university. In summary, future studies will need more representative samples that cover emerging adults from a diversity of educational, social, and cultural backgrounds and with a greater balance in gender.

Another main area of limitation is the cross-sectional design of this study, thus, causal inferences cannot be made, while future research on this topic would benefit from the inclusion of longitudinal studies. Finally, while the Five Cs were largely reported among the participants, there is still the question as to whether the Five Cs actually reflect positive indicators for Romanian emerging adults. For example, Geldhof et al. (2015) reasoned that the Five Cs measures were created to capture broad aspects of PYD indicators that are relevant to most young people, but the measures may still lack content specificity. Accordingly, the meaning of each C may differ across context. It is, therefore, possible that the Five Cs measures that were developed using youth samples living in a WEIRD national context do not only fail to accurately assess the competence, caring, confidence, connection, and character of the participants who live in a non-WEIRD context, but also that their indicators of positive development are not adequately captured by the measures. These are issues that need to be examined in future studies that will also utilize qualitative methods. Greater diversity in research methods, such as fully ideographic individual focused research over time, should be used to investigate this topic as that would provide a fuller test of the specificity principle (Bornstein, 2017) as it relates to the identification of universal as well as possibly culturally specific profiles of positive psychological strengths.

### Implications

Despite the limitations, the findings have several implications related to research, policy, and practice. For research, the findings extend the applicability of the Five Cs of PYD scale and indicate that the scale can be used as a tool in accessing indicators of positive psychological strengths, also among emerging adults attending university in Romania, although some refinement and exploration of a wider set of constructs may be required. With the Five Cs measure as a starting point, more research can be conducted to fully assess the facilitating factors of thriving among youth and emerging adults in Romania, and similar contexts in Eastern Europe where PYD research is scarce. Applying a strengths-based framework in strategies that are put together to promote the course of the youth and emerging adults may constitute an effective way of harnessing and channeling their strengths and skills into community and national development. Accordingly, stakeholders at all levels of society in the Romanian contexts can have it as a policy to promote a coordinated effort that ensures nurturance of the skills, opportunities, and resources needed for thriving and contribution among Romanian youth and emerging adults.

As for practice, the Five Cs can be actively used in intervention settings, not just as a framework but also as measures that provide an indication of the level of psychological strengths. Using PYD measures as an assessment of positive development will help to identify the needs and strengths of the youth and emerging adults as well as to help determine where to invest more resources. For profiles of youth and emerging adults with higher levels of psychological strengths, such as in Class 2, intervention strategies that will sustain these levels can be applied. For profiles in which there is substantial room to grow in terms of the levels of strengths, for example character and caring in Class 1, interventions that will enhance the psychological strengths could be particularly beneficial. Thus, specific components of the Five Cs can be focused on in intervention programs depending on the needs of the group or individuals. Moreover, as PYD proposes that positive psychological strengths are a function of an active interaction between individuals and their contexts, emerging adults should be given the opportunity to participate actively in the planning and implementation of programs that concern their own development and well-being (Eichas et al., 2019).

### Conclusion

Results indicated two profiles, Class 2 that showed above average to high levels of the Five Cs, and Class 1, a pattern that contains low to above average scores, with lower scores on character and caring. Although Class 1 was less likely to report somatic complaints, Class 2 was positively associated with other indicators of well-being. These are important findings, not only because of the contribution to the generalizability of the Five Cs measures and theory but also because of the implications of the findings to research, policy, and practice in the Romanian contexts and beyond. The findings show indications of thriving among emerging adults attending university in the Romanian context, but there is much more work to be done.

## Data Availability Statement

The data for this study is not publicly available because this study's ethical review does not allow for the primary data to be in a public repository. Individual data requests for de-identified (IPD) will be reviewed for qualified researchers (e.g., Ph.D.) who obtain ethical permission under Norwegian ethical regulations/laws for secondary data analysis for purposes such as meta-analysis or confirmation of published study results. Requests to access the IPD datasets should be directed to Laura Ferrer-Wreder, laura.ferrer-wreder@psychology.su.se.

## Ethics Statement

This study was reviewed and approved by the NSD - Norwegian Center for Research Data as part of the cross-national study of positive development. Written informed consent for study participation was not required for this study in accordance with the national legislation and institutional requirements.

## Author Contributions

LF-W led the overall writing of this article. KE conducted the analysis and provided the interpretation of the data. CB and DS conducted the data collection and formulated the study design. NW led the writing of the discussion section, obtained ethical approval from Norwegian ethical review and leads the Cross-National Study of Positive Development, in which this study is embedded. All authors made substantial contributions to the design and drafting of this article, gave their approval for the publication of this article and its content, and agreed to be accountable for attesting to the accuracy of the study represented in this article.

## Conflict of Interest

The handling editor is currently organizing a Research Topic with the authors LF-W and NW. The remaining authors declare that the research was conducted in the absence of any commercial or financial relationships that could be construed as a potential conflict of interest.

## Footnotes

^1^The brackets in this quote were added by the authors of this study to convey the full meaning of the quote, which is part of a longer discussion by the quoted authors about the merit and pitfalls of variable and person-oriented research in the study on positive youth development.

## References

[B1] Arbeit M. R. Johnson S. K. Champine R. B. Greenman K. N. Lerner J. V. Lerner R. M. (2014). Profiles of problematic behaviors across adolescence: covariations with indicators of positive youth development. J. Youth Adolesc. 43, 971–990. 10.1007/s10964-014-0092-0 24562425

[B2] Asparouhov T Muthén B. (2012). Auxiliary Variables in Mixture Modeling: A 3-Step Approach Using Mplus. Mplus Web Notes No. 2012 15 (Version 5).

[B3] Bădescu G. Sandu D. Angi D. Greab C. (2019). Youth Study Romania 2018/2019. Available online at http://library.fes.de/pdf-files/id-moe/15268.pdf (accessed January 26, 2021).

[B4] Bădescu G. Sum P. (2015). Generalized trust and diversity in the classroom: a longitudinal study of Romanian adolescents. Commun. Post-Commun. Stud. 48, 33–41. 10.1016/j.postcomstud.2015.01.004

[B5] Bergman L. R. Andersson H. (2010). The person and the variable in developmental psychology. J. Psychol. 218, 155–165. 10.1027/0044-3409/a000025

[B6] Bornstein M. H. (2017). The specificity principle in acculturation science. Perspect. Psychol. Sci. 12, 3–45. 10.1177/1745691616655997 28073331 PMC5234695

[B7] Conway R. J. Heary C. Hogan M. J. (2015). An evaluation of the measurement properties of the five Cs model of positive youth development. Front. Psychol. 6:1941. 10.3389/fpsyg.2015.01941 26733923 PMC4686649

[B8] Cosma A. Stevens G. Martin G. Duinhof E. L. Walsh S. D. Garcia-Moya I.. (2020). Cross-national time trends in adolescent mental well-being from 2002 to 2018 and the explanatory role of schoolwork pressure. J. Adolesc. Health 66, S50–S58. 10.1016/j.jadohealth.2020.02.010 32446609 PMC8131201

[B9] David D. (2015). Psihologia poporului român. Profilulpsihologic al românilorîntr-o monografiecognitivexperimentală [Psychology of the Romanian people. The psychological profile of Romanians in a cognitive-experimental monograph]. Iaşi: Polirom

[B10] Diamond L. (2015). Facing up to the democratic recession. J. Democracy 26, 141–155. 10.1353/jod.2015.0009

[B11] Dimitrova R. Musso P. Abubakar A. Šolcová I. Stefenel D. Uka F.. (2017). Identity resources for positive adaptation of Roma ethnic minority youth in Albania, Bulgaria, the Czech Republic, Italy, Kosovo, and Romania in Positive Youth Development in Global Contexts of Social and Economic Change, eds. Petersen A. Koller S.H. Motti-Stefanidi F. Verma S. (New York, NY: Taylor and Francis), 184–199.

[B12] Eichas K. Ferrer-Wreder L. Olsson T. M. (2019). Contributions of positive youth development to intervention science. Child Youth Care Forum 48, 279–287. 10.1007/s10566-018-09486-1

[B13] European Commission (2019). Education and Training Monitor Romania. Available online at: https://ec.europa.eu/education/sites/education/files/document-library-docs/et-monitor-report-2019-romania_en.pdf (accessed January 26, 2021).

[B14] European Monitoring Center for Drugs Drug Addiction (2019). Romania Country Drug Report. Available online at: https://www.emcdda.europa.eu/countries/drug-reports/2019/romania/drug-use_en (accessed January 26, 2021).

[B15] Eurostat (2019). Young People Neither in Employment Nor in Education and Training by Sex, Age and Degree of Urbanisation (NEET Rates). Available online at: https://appsso.eurostat.ec.europa.eu/nui/show.do?dataset=edat_lfse_29andlang=en (accessed January 26, 2021).

[B16] Eurostat (2020). Population by Educational Attainment Level, Sex and Age (%). Available online at: http://appsso.eurostat.ec.europa.eu/nui/submitViewTableAction.do (accessed January 26, 2021).

[B17] Geldhof G. J. Bowers E. P. Boyd M. J. Mueller M. Napolitano C. M. Schmid K. L.. (2014a). The creation and validation of short and very short measures of PYD. J. Res. Adolesc. 24, 163–176. 10.1111/jora.12039

[B18] Geldhof G. J. Bowers E. P. Mueller M. K. Napolitano C. M. Callina K. S. Lerner R. M. (2014b). Longitudinal analysis of a very short measure of positive youth development. J. Youth Adolesc. 43, 933–949. 10.1007/s10964-014-0093-z 24557779

[B19] Geldhof G. J. Bowers E. P. Mueller M. K. Napolitano C. M. Callina K. S. Walsh K. J.. (2015). The five Cs model of positive youth development, in Promoting Positive Youth Development, eds Bowers E. P. Geldhof G. J. Johnson S. K. Hilliard L. J. Hershberg R. M. Lerner J. V. Lerner R. M. (Cham: Springer Verlag), 161–186.

[B20] Henrich J. Heine S. J. Norenzayan A. (2010). The weirdest people in the world? Behav. Brain Sci. 33, 61–83. 10.1017/S0140525X0999152X 20550733

[B21] Herth K. (1991). Development and refinement of an instrument to measure hope. Sch. Inq. Nurs. Pract. 5, 39–51. 2063043

[B22] Hill P. L. Edmonds G. W. Peterson M. Luyckx K. Andrews J. A. (2016). Purpose in life in emerging adulthood: development and validation of a new brief measure. J. Positive Psychol. 11, 237–245. 10.1080/17439760.2015.1048817 26958072 PMC4779362

[B23] Hofstede G. (2020). Romania. Available online at: https://www.hofstede-insights.com/country-comparison/romania/ (accessed January 26, 2021).

[B24] Holsen I. Geldhof J. Larsen T. Aardal E. (2017). The Five Cs of positive youth development in Norway: assessment and associations with positive and negative outcomes. Int. J. Behav. Dev. 41, 559–569. 10.1177/0165025416645668

[B25] Hu L. Bentler P. M. (1999). Cutoff criteria for fit indexes in covariance structure analysis: conventional criteria versus new alternatives. Struct. Equ. Model. 6, 1–55. 10.1080/10705519909540118

[B26] Inchley J. Currie D. Cosma A.. (2018). Health Behavior in School-Aged Children (HBSC) Study Protocol: Background, Methodology and Mandatory Items for the 2017/18 Survey. St. Andrews: CAHRU.

[B27] Karaś D. Cieciuch J. Negru O. Crocetti E. (2014). Relationships between identity and well-being in Italian, Polish, and Romanian Emerging Adults. Soc. Indic. Res. 121, 727–743. 10.1007/s11205-014-0668-9

[B28] Keyes C. L. M. Yao J. Hybels C. F. Milstein G. Proeschold-Bell R. (2020). Are changes in positive mental health associated with increased likelihood of depression over a two-year period? A test of the mental health promotion and protection hypotheses. J. Affect. Disord. 270, 136–142. 10.1016/j.jad.2020.03.056 32339105

[B29] Leffert N. Benson P. L. Scales P. C. Sharma A. R. Drake D. R. Blyth D. A. (1998). Developmental assets: measurement and prediction of risk behaviors among adolescents. Appl. Dev. Sci. 2, 209–230. 10.1207/s1532480xads0204_4

[B30] Lerner R. M. Lerner J. V. Almerigi J. B. Theokas C. Phelps E. Gestsdottir S.. (2005). Positive youth development, participation in community youth development programs, and community contributions of fifth-grade adolescents: findings from the first wave of the 4-H Study of Positive Youth Development. J. Early Adolesc. 25, 17–71. 10.1177/0272431604272461

[B31] Lerner R. M. Tirrell J. M. Dowling E. M. Geldhof G. J. Gestsdóttir S. Lerner J. V.. (2019). The end of the beginning: Evidence and absences studying positive youth development in a global context. Adolesc. Res. Rev. 4, 1–14. 10.1007/s40894-018-0093-4

[B32] Lewin-Bizan S. Lynch A. D. Fay K. Schmid K. McPherran C. Lerner J. V.. (2010). Trajectories of positive and negative behaviors from early- to middle-adolescence. J. Youth Adolesc. 39, 751–763. 10.1007/s10964-010-9532-7 20387107

[B33] Little T. D. Rhemtulla M. Gibson K. Schoemann A. M. (2013). Why the items versus parcels controversy needn't be one. Psychol. Methods 18, 285–300. 10.1037/a0033266 23834418 PMC3909043

[B34] Lyyra N. Välimaa R. Tynjälä J. (2018). Loneliness and subjective health complaints among school-aged children. Scand. J. Public Health 46, 87–93. 10.1177/1403494817743901 29552967

[B35] Magnusson D. (2001). The holistic-interactionistic paradigm: some directions for empirical developmental research. Eur. Psychol. 6, 153–162. 10.1027//1016-9040.6.3.153

[B36] Negru O. (2012). The time of your life: emerging adulthood characteristics in a sample of Romanian high-school and university students. Cogn. Brain Behav. 16, 357−367

[B37] NIS [National Institute of Statistics] (2013). Ce ne spunerecensământul din 2011 despre RELIGIE? [What does the 2011 census tell us about Religion?]. Available online at http://www.insse.ro/cms/files/publicatii/pliante%20statistice/08-%20Recensamintele%20despre%20religie_n.pdf (accessed January 26, 2021).

[B38] Nylund-Gibson K. Choi A. Y. (2018). Ten frequently asked questions about latent class analysis. Transl. Issues Psychol. Sci. 4, 440–461. 10.1037/tps0000176

[B39] O'Connor M. Sanson A. V. Toumbourou J. W. Hawkins M. T. Letcher P. Williams P.. (2016). Positive development and resilience in emerging adulthood, in The Oxford Handbook of Emerging Adulthood, ed Arnett J. J. (New York, NY: Oxford University Press), 601–614.

[B40] Overton W. F. (2015). Process and relational developmental systems, in Handbook of Child Psychology and Developmental Science, eds, Overton W. F. Molenaar P. C. M. Lerner R. M. Editor-in-Chief, (Hoboken, NJ: Wiley), 9-62.

[B41] Rada C. Ispas A. T. (2016). Alcohol consumption and accentuated personality traits among young adults in Romania: a cross-sectional study. Subst. Abuse Treatm. Prev. Policy 11, 11–36. 10.1186/s13011-016-0080-3 27784325 PMC5081981

[B42] Romanian National Statistics Institute (2019). Social Trends. Available online at https://insse.ro/cms/sites/default/files/field/publicatii/tendinte_sociale.pdf (accessed January 26, 2021).

[B43] Schwartz S. H. (2008). Cultural Value Orientations: Nature and Implications of National Differences. Moscow: State University—Higher School of Economics Press.

[B44] Seligman M. E. Csikszentmihalyi M. (2000). Positivepsychology: an introduction. Am. Psychol. 55, 5–14. 10.1007/978-94-017-9088-8_18 11392865

[B45] Tolan P. Ross K. Arkin N. Godine N. Clark E. (2016). Toward an integrated approach to positive development: implications for intervention. Appl. Dev. Sci. 20, 214–236. 10.1080/10888691.2016.1146080

[B46] Truskauskaite-Kunevičiene I. Žukauskaine R. Kaniušonyte G. (2014). Positive youth development links to satisfaction with life, resilience and internalizing and externalizing problems. Social Work 13, 98–109. 10.13165/SD-14-13-1-09

[B47] van de Vijver F. (2019). Cross-cultural research, in Advanced Research Methods for the Social and Behavioral Sciences, eds Edlundand J. Nichols A. (London, England: Cambridge University Press), 274–286.

[B48] van Gestel-Timmermans H. van den Bogaard J. Brouwers E. Herth K. van Nieuwenhuizen C. (2010). Hope as a determinant of mental health recovery: a psychometric evaluation of the Herth hope index-Dutch version. Scand. J. Car. Sci. 24, 67–74. 10.1111/j.1471-6712.2009.00758.x 20210899

[B49] Vincze A. E. Csaba D. Roth M. Hărăguş T. P. (2013). A nationwide study of mental health and social support among Romanian adolescents transitioning to adulthood. ErdélyiPszichológiaiSzemle 14, 93–122.

[B50] Wiium N. Dimitrova R. (2019). Positive youth development across cultures. Introduction to the special issue. Child Youth Care Forum 48, 147–153. 10.1007/s10566-019-09488-7

[B51] Wiium N. Ferrer-Wreder L. Chen B-B. Dimitrova R. (2019). Gender and positive youth development advancing sustainable development goals in Ghana. Z. Psychol. 227, 134–138. 10.1027/2151-2604/a000365

[B52] World Health Organization (2018). Global status report on alcohol and health 2018. Available online at https://movendi.ngo/wp-content/uploads/2019/11/9789241565639-eng.pdf (accessed January 26, 2021).

[B53] World Health Organization (2020). Health behaviours among adolescents in Romania: Health Behaviour in School-aged Children (HBSC) study 2018, Research Report. Available online at https://drive.google.com/file/d/1_RITbl7uxKIGjhjFNLv6Vi3fOmM08kyI/view (accessed January 26, 2021).

