# Supplementary material for: The Importance of Positive Psychological Strengths in Well-Being and Adjustment of Romanian Emerging Adults: A Pattern and Variable-Oriented Approach
Source: Front Psychol. 2021 Jul 21;12:659300. doi: 10.3389/fpsyg.2021.659300 (PMC8335555; doi:10.3389/fpsyg.2021.659300)
Supplement: Supplementary file 1 [file Data_Sheet_1.docx]

**Supplementary Material (SM)**

**Appendix 1**

**Details of Measurement Analyses Conducted in this article.**

For all measurement analyses, we used a mean- and variance-adjusted weighted least squares estimator (Mplus’s WLSMV estimator) to accommodate the ordinal nature of the item-level data.

The hypothesized measurement model for the Purpose in Life short form was a one-factor model (Hill et al., 2016). As shown in SM Table 1, the hypothesis of exact fit was ruled out based on a statistically significant chi square value (p < .001). The hypothesized model provided acceptable fit according to the CFI (.995), TLI (.985), and SRMR (.019), but not the RMSEA (.210). However, because there were no theoretically supported modifications available, the one-factor model was retained. The factor loadings from this model are presented in SM Table 2.

The hypothesized measurement model for the Herth Hope Index was also a one-factor model. Because there were two reverse coded items, an error covariance for those two items was specified. As shown in SM Table 1, the hypothesized model failed to provide acceptable fit. We explored possible modifications that would improve model fit by examining modification indices. Based on a modification index of 285.75, we added an error covariance for items 9 and 10. Both of these items were statements about caring/love (SM Table 3). The modified model also failed to yield acceptable fit. Therefore, we trimmed eight items with standardized factor loadings of less than .60. See SM Table 3 for a list of the retained and trimmed items. Trimming these eight items yielded a model that passed the exact fit test and provided acceptable fit according to the CFI, TLI, SRMR, and RMSEA. This trimmed one-factor model with five items (items 1, 2, 11, 12, and 13 in SM Table 3) was retained. The factor loadings from this model are presented in SM Table 3.

Two measurement models for the Subjective Health Complaints scale from the Health Behaviour in School-aged Children (HBSC) survey were tested: a two-factor model (psychological complaints and somatic complaints; Gariepy, McKinnon, Sentenac, & Elgar, 2016) and a more parsimonious one-factor model. As shown in SM Table 1, the hypothesis of exact fit for the two-factor model was ruled out because of a significant chi square value. The model provided acceptable fit according to the CFI, TLI, and SRMR, but not the RMSEA. The one-factor model provided significantly worse model fit, χ2Wald (1) = 23.92, p < .001. Next, we explored possible modifications that would improve the fit of the two-factor model by examining modification indices. Based on a modification index of 19.51, we added an error covariance for items 4 and 6 to reflect the hypothesis that causes of depressed mood would decrease feelings of nervousness, and vice versa. The modified model yielded acceptable fit. The factor loadings from this model are presented in SM Table 4.

Three measurement models for the Mental Health Continuum Short Form were tested: a one-factor model (general well-being), a three-factor model (emotional, social, and psychological well-being), and a bifactor model that included both the general well-being factor and the specific well-being factors (Jovanovic, 2015). As shown in SM Table 1, the one-factor model did not provide acceptable fit. The three-factor model provided acceptable fit according to the CFI and SRMR but not the TLI or RMSEA. We selected the bifactor model because, although it did not provide exact fit, it met cutoff criteria for the CFI, TLI, RMSEA, and SRMR. Examination of the factor loadings from the bifactor model revealed several nonsignificant loadings. These loadings were trimmed from the model, but the items were retained as indicators of the general well-being factor. This modified bifactor model also provided acceptable fit and was retained. The factor loadings from this model are presented in SM Table 5.

The hypothesized measurement model for the PYD Short Form was a five-factor model. We trimmed six items from the model that had performed poorly in past research (Tirrell et al., 2019): two physical competence items (competence in athletics/sports), two physical appearance items (confidence), and two conduct items (character). See SM Table 6 for the retained and trimmed items. Although model fit improved, it still did not meet criteria for acceptable fit. Next, we explored possible modifications that would improve model fit by examining modification indices. Based on modification indices ranging from 41.90 to 197.014, we added an error covariances for pairs of items with overlapping content including friendship (items 33 and 34), valuing diversity (items 19 and 20), family (items 28 and 31) and connection to university (items 27 and 30). Adding these error covariances yielded a model that provided acceptable fit according to the CFI, TLI, and RMSEA, but not the SRMR. Because there were no further theoretically supported modifications available, the modified five-factor model was retained. Factor loadings from this model are presented in SM Table 6.

The hypothesized measurement models for both Substance Use/Abuse and Antisocial Behavior were one-factor models. As shown in SM Table 1, the hypothesized Substance Use/Abuse model provided good fit, whereas the hypothesized Antisocial Behavior model yielded an inadmissible solution, suggesting model misspecification. One item (skipped school/lectures) was trimmed from the model because it was negatively correlated with other items. The modified model yielded acceptable fit according to the chi square p-value, CFI, TLI, and RMSEA.

| **Supplementary Material (SM) Tables**  SM Table 1. Model Fit, Measurement Models | | | | | | | |
| --- | --- | --- | --- | --- | --- | --- | --- |
|  | *χ*^2^(df) | *p* | CFI | TLI | RMSEA | CFIT | SRMR |
| Purpose in Life |  |  |  |  |  |  |  |
| **Hypothesized 1-factor** | 26.057(2) | < .001 | .995 | .985 | .210[.143, .286] | < .001 | .019 |
| Hope |  |  |  |  |  |  |  |
| Hypothesized 1-factor | 536.566(64) | < .001 | .807 | .765 | .165[.152, .178] | < .001 | .095 |
| Modified 1-factor | 277.052(63) | < .001 | .913 | .892 | .112[.098, .125] | < .001 | .068 |
| **Trimmed 1-factor** | 10.068(5) | .073 | .996 | .992 | .061[.000, .116] | .309 | .015 |
|  |  |  |  |  |  |  |  |
| Health Behavior (Subjective Health Complaints) | | | | | | | |
| Hypothesized 2-factor | 70.178(19) | < .001 | .969 | .955 | .100[.075, .125] | .001 | .042 |
| Hypothesized 1-factor | 104.646(20) | < .001 | .949 | .929 | .125[.102, .149] | < .001 | .052 |
| **Modified 2-factor** | 49.954(18) | < .001 | .981 | .970 | .081[.055, .108] | .028 | .037 |
|  |  |  |  |  |  |  |  |
| Well-Being (Mental Health Continuum Short Form) | | | | | | | |
| Hypothesized 1-factor | 700.261(77) | < .001 | .844 | .816 | .173[.161, .184] | < .001 | .078 |
| Hypothesized 3-factor | 278.858(74) | < .001 | .950 | .938 | .100[.088, .113] | < .001 | .049 |
| Hypothesized Bifactor | 157.258(60) | < .001 | .976 | .963 | .077[.063, .092] | .002 | .033 |
| **Modified Bifactor** | 153.124(64) | < .001 | .978 | .968 | .072[.057, .086] | .008 | .033 |
|  |  |  |  |  |  |  |  |
| Positive Youth Development Short Form (PYDSF) | | | | | | | |
| Hypothesized 5-factor | 1630.963(517) | < .001 | .845 | .832 | .089[.084, .094] | < .001 | .096 |
| Trimmed 5-factor | 942.294(340) | < .001 | .911 | .901 | .081[.075, .087] | < .001 | .083 |
| **Modified 5-factor** | 628.655(336) | < .001 | .957 | .951 | .057[.050, .063] | .057 | .066 |
|  |  |  |  |  |  |  |  |
| Substance Use/Abuse | | | | | | | |
| **Hypothesized 1-factor** | 13.884(14) | .458 | 1.000 | 1.000 | .000[.000, .058] | .897 | .068 |
| Antisocial Behavior | | | | | | | |
| Hypothesized 1-factor | -- | -- | -- | -- | -- | -- | -- |
| **Modified 1-factor** | 9.281(5) | .098 | .978 | .956 | .056[.000, .112] | .362 | .100 |
|  |  |  |  |  |  |  |  |

*Notes*. Text in **Bold** above indicates model retained for further analyses

| SM Table 2. Parameter Estimates, Purpose in Life |  |
| --- | --- |
| Items | Est. (95% CI) |
|  |  |
| 1. There is a direction in my life. | .921 [.893, .949] |
| 2. My plans for the future match with my true interests and values. | .853 [.815, .891] |
| 3. I know which direction I am going to follow in my life. | .953 [.930, .976] |
| 4. My life is guided by a set of clear commitments. | .790 [.739, .841] |
|  |  |
| *Composite Reliability* |  |
| McDonald’s *ω* | .933 [.930, .936] |
|  |  |

| SM Table 3. Parameter Estimates, Hope |  |
| --- | --- |
| Items | Est. (95% CI) |
|  |  |
| 1. I have a positive outlook toward life. | .691 [.616, .766] |
| 2. I have short and/or long range goals. | .632 [.547, .717] |
| 3. I feel all alone. (r) | -- |
| 4. I can see possibilities in the midst of difficulties. | -- |
| 5. I have a faith that gives me comfort. | -- |
| 6. I feel scared about my future. (r) | -- |
| 7. I can recall happy/joyful times. | -- |
| 8. I have deep inner strength. | -- |
| 9. I am able to give caring/love. | -- |
| 10. I am able to receive caring/love. | -- |
| 11. I have a sense of direction. | .823 [.769, .877] |
| 12. I believe that each day has potential. | .806 [.750, .862] |
| 13. I feel that my life has value and worth. | .846 [.793, .900] |
|  |  |
| *Composite Reliability* |  |
| McDonald’s *ω* | .874 [.864, .884] |
|  |  |

*Notes.* Items trimmed from the final retained measurement model were items 3, 4, 5, 6, 7, 8, 9, 10 listed in Table 3 (above).

| SM Table 4. Parameter Estimates, Health Behavior (Subjective Health Complaints) | | |
| --- | --- | --- |
|  | Est. (95% CI) | |
| Items | Somatic | Psychological |
|  |  |  |
| 1. Headache | .621 [.521, .720] |  |
| 2. Stomach ache | .614 [.515, .713] |  |
| 3. Backache | .536 [.423, .649] |  |
| 8. Dizziness | .828 [.740, .916] |  |
| 4. Felt low/depressed |  | .881 [.817, .946] |
| 5. Felt irritable/Bad tempered |  | .808 [.749, .867] |
| 6. Felt nervous |  | .849 [.786, .913] |
| 7. Difficulty sleeping |  | .515 [.420, .610] |
|  |  |  |
| *Factor Correlation* |  |  |
| Somatic |  | .769 [.685, .853] |
|  |  |  |
| *Error Covariance* |  |  |
| Item 4 🡨🡪 Item 6 |  | -.937 [-1.535, -.339] |
|  |  |  |
| *Composite Reliability* |  |  |
| McDonald’s *ω* | .749 [.721, .778] | .893 [.876, .910] |
|  |  |  |

| SM Table 5. Parameter Estimates, Well-Being (Mental Health Continuum Short Form) | | | | |
| --- | --- | --- | --- | --- |
|  | Est. (95% CI) | | | |
| ***Items*** | General | Emotional | Social | Psychological |
| 1. Happy | .563 [.478, .648] | .608 [.516, .700] |  |  |
| 2. Interested in life | .690 [.621, .760] | .517 [.430, .604] |  |  |
| 3. Satisfied | .612 [.529, .695] | .640 [.544, .736] |  |  |
| 4. That you had something important to contribute to society | .465 [.365, .565] |  | .384 [.286, .481] |  |
| 5. That you belonged to a community (like a social group, school, neighborhood, etc.) | .621 [.546, .696] |  | .327 [.238, .416] |  |
| 6. That society is a good place, or is becoming a better place, for all people | .475 [.381, .568] |  | .738 [.670, .806] |  |
| 7. That people are basically good | .434 [.332, .536] |  | .670 [.596, .806] |  |
| 8. That the way society works made sense to you. | .378 [.275, .481] |  | .727 [.650, .805] |  |
| 9. That you liked most parts of your personality. | .689 [.617, .760] |  |  | .706 [.426, .987] |
| 10. Good at managing the responsibilities of your daily life. | .684 [.615, .752] |  |  | .301 [.157, .445] |
| 11. That you had warm and trusting relationships with others. | .677 [.604, .750] |  |  |  |
| 12. That you had experiences that challenged you to grow and become a better person. | .675 [.604, .746] |  |  |  |
| 13. Confident to think or express your own ideas and opinions. | .734 [.671, .796] |  |  |  |
| 14. That your life has a sense of direction or meaning to it. | .465 [.769, .882] |  |  |  |
| ***Factor Correlations*** | | | | |
| General | -- | -- | -- | -- |
| Emotional |  | -- | .352 [.208, .496] | .154 [-.007, .314] |
| Social |  |  | -- | .328 [.144, .512] |
| Psychological |  |  |  | -- |
| ***Composite Reliability*** |  |  |  |  |
| McDonald’s *ω* | .934 [.925, .943] | .798 [.766, .831] | .797 [.770, .824] | .684 [.594, .773] |
|  |  |  |  |  |

*Notes.* All fourteen items were retained in the final measurement model, but the loadings of items 11-14 on the specific psychological well-being factor.

| SM Table 6. Parameter Estimates, Positive Youth Development Short Form | | | | |  |
| --- | --- | --- | --- | --- | --- |
|  | Esttimate (95% CI) | | | | |
| Items | Competence | Confidence | Character | Caring | Connection |
|  |  |  |  |  |  |
| 1. I have a lot of friends. | .683 [.597, .770] |  |  |  |  |
| 2. I do very well in my assignments at the university. | .424 [.311, .537] |  |  |  |  |
| 7. I am just as smart as others my age. | .399 [.289, .509] |  |  |  |  |
| 9. I am popular with others my age. | .650 [.567, .734] |  |  |  |  |
| 4. I am happy with myself most of the time. |  | .691 [.613, .769] |  |  |  |
| 12. I am very happy being the way I am. |  | .918 [.882, .955] |  |  |  |
| 13. All in all, I am glad I am me. |  | .920 [.888, .953] |  |  |  |
| 14. When I am an adult, I am sure I will have a good life. |  | .696 [.618, .774] |  |  |  |
| 15. Helping to make the world a better place to live in. |  |  | .512 [.391, .633] |  |  |
| 16. Accepting responsibility for my actions when I make a mistake or get into trouble |  |  | .488 [.370, .606] |  |  |
| 17. Giving time and money to make life better for other people. |  |  | .612 [.518, .706] |  |  |
| 18. Doing what I believe is right even when my friends make fun of me. |  |  | .551 [.444, .659] |  |  |
| 19. Happy being with others who are of different ethnicity/culture than I am. |  |  | .582 [.470, .695] |  |  |
| 20. Knowing a lot about people of other ethnicities/culture. |  |  | .686 [.585, .787] |  |  |
| 21. When I see people being taken advantage of, I want to help them. |  |  |  | .808 [.757, .860] |  |
| 22. When I see someone being picked on, I feel sorry for them. |  |  |  | .887 [.841, .932] |  |
| 23. When I see another person who is hurt or upset, I feel sorry for them. |  |  |  | .868 [.825, .910] |  |
| 24. It bothers me when bad things happen to any person. |  |  |  | .768 [.705, .832] |  |
| 25. I feel sorry for other people who don’t have what I have. |  |  |  | .776 [.718, .835] |  |
| 26. It makes me sad to see a person who doesn’t have friends. |  |  |  | .725 [.660, .790] |  |
| 27. I receive a lot of encouragement at my department/university. |  |  |  |  | .469 [.371, .567] |
| 28. I am a useful and important member of my family. |  |  |  |  | .649 [.545, .753] |
| 29. I feel like an important member of my local community. |  |  |  |  | .721 [.643, .798] |
| 30. Teachers at the department/university to be the best that I can be. |  |  |  |  | .488 [.390, .586] |
| 31. I have lots of good conversation with my parents. |  |  |  |  | .461 [.346, .577] |
| 32. Adults in my town/city/local community listen to what I have to say. |  |  |  |  | .621 [.527, .715] |
| 33. My friends care about me. |  |  |  |  | .568 [.476, .660] |
| 34. I feel my friends are good friends. |  |  |  |  | .494 [.391, .598] |
|  |  |  |  |  |  |
| *Factor Correlations* |  |  |  |  |  |
| Competence | -- | .733 [.640, .825] | .238 [.088, .388] | .138 [-.012, .288] | .763 [.670, .855] |
| Confidence |  | -- | .182 [.050, .314] | .014 [-.115, .142] | .661 [.574, .748] |
| Character |  |  | -- | .802 [.724, .879] | .380 [.260, .500] |
| Caring |  |  |  | -- | .230 [.104, .357] |
| Connection |  |  |  |  | -- |
|  |  |  |  |  |  |
| *Composite Reliability* |  |  |  |  |  |
| McDonald’s *ω* | .627[.579, .674] | .912[.905, .919] | .707[.662, .751] | .921[.916, .926] | .714[.674, .753] |
|  |  |  |  |  |  |

*Notes.* Items trimmed from the retained model (not shown in the SM Table 6) were: two competence items (I am better than others my age at sports and I could do well at just any athletic/sport activity), two confidence items (I really like the way I look and I am good looking), and two Character items (I hardly ever do things I know I shouldn't do and I usually act the way am supposed to).

| SM Table 7. Parameter Estimates, Substance Use/Abuse |  |
| --- | --- |
| Items | Est. (95% CI) |
|  |  |
| 1. Have you used alcohol once or more in the past 30 days? | .654 [.482, .825] |
| 2. Have you been drunk once or more in the past 30 days? | .644 [.493, .794] |
| 3. Have you smoked cigarettes once or more in the past 30 days? | .772 [.659, .885] |
| 4. Have you sniffed or inhaled substances to get high once or more in the last 12 months? | .965 [.849, 1.081] |
| 5. Have you used other illicit drugs (e.g., cocaine, LSD, heroin, amphetamines, etc.) once or more in the last 12 months? | .911 [.775, 1.048] |
| 6. Have you driven after drinking, once or more in the last 12 months? | .654 [.456, .853] |
| 7. Have you been a passenger (once or more in the last 12 months) with a driver who has been drinking? | .251 [.034, .467] |
|  |  |
| *Composite Reliability* |  |
| McDonald’s *ω* | .877 [.857, .896] |
|  |  |

| SM Table 8. Parameter Estimates, Antisocial Behavior |  |
| --- | --- |
| Items | Est. (95% CI) |
|  |  |
| 1. Have you been involved in shop lifting once or more in the last 12 months? | .981 [.690, 1.272] |
| 2. Have you committed vandalism once or more in the last 12 months? | .607 [.449, .765] |
| 3. Have you been in a group fight once or more in the last 12 months? | .563 [.435, .692] |
| 4. Have you carried a weapon for protection once or more in the last 12 months? | .596 [.418, .774] |
| 5. Have you skipped school/lectures once or more in the last 4 weeks? | -- |
| 6. Have you gambled with money once or more in the last 12 months? | .410 [.269, .551] |
|  |  |
| *Composite Reliability* |  |
| McDonald’s *ω* | .779 [.740, .818] |
|  |  |

| **Appendix 2**  **Supplementary Analyses: Factor Correlations in this Sample** | | | | | | |  |
| --- | --- | --- | --- | --- | --- | --- | --- |
| Factors | 1. | 2. | 3. | 4. | 5. | 6. | |
|  |  |  |  |  |  |  | |
| 1. Competence | -- | .736 [.615, .857] | .200 [.040, .361] | .175 [.001, .349] | .628 [.506, .750] | .766 [.661, .871] | |
| 2. Confidence |  | -- | .118 [-.033, .269] | .034 [-.120, .188] | .605 [.487, .722] | .783 [.698, .869] | |
| 3. Character |  |  | -- | .661 [.561, .761] | .287 [.141, .433] | .317 [.172, .461] | |
| 4. Caring |  |  |  | -- | .226 [.076, .376] | .169 [.010, .327] | |
| 5. Connection |  |  |  |  | -- | .749 [.653, .844] | |
| 6. Wellbeing |  |  |  |  |  | -- | |
| 7. Hope |  |  |  |  |  |  | |
| 8. Purpose |  |  |  |  |  |  | |
| 9. Somatic Complaints |  |  |  |  |  |  | |
| 10. Psychological Complaints |  |  |  |  |  |  | |
| 11. Substance Abuse |  |  |  |  |  |  | |
| 12. Antisocial Behavior |  |  |  |  |  |  | |
|  |  |  |  |  |  |  | |
| Factors | 7. | 8. | 9. | 10. | 11. | 12. | |
|  |  |  |  |  |  |  | |
| 1. Competence | .652 [.536, .767] | .569 [.448, .689] | -.131 [-.317, .056] | -.448 [-.603, -.293] | -.051 [-.235, .132] | .082 [-.070, .234] | |
| 2. Confidence | .806 [.732, .880] | .641 [.544, .737] | -.210 [-.359, -.062] | -.409 [-.543, -.274] | -.082 [-.219, .054] | .086 [-.045, .218] | |
| 3. Character | .251 [.092, .410] | .299 [.155, .442] | .229 [.079, .379] | -.023 [-.180, .134] | .016 [-.123, .156] | .112 [-.021, .245] | |
| 4. Caring | .154 [-.010, .318] | .145 [-.005, .295] | .310 [.172, .447] | .213 [.073, .353] | .038 [-.079, .155] | .058 [-.057, .173] | |
| 5. Connection | .559 [.411, .708] | .465 [.322, .607] | -.118 [-.276, .040] | -.334 [-.474, -.193] | -.137 [-.272, -.002] | -.009 [-.163, .146] | |
| 6. Wellbeing | .815 [.737, .892] | .692 [.603, .781] | -.101 [-.254, .051] | -.479 [-.617, -.341] | -.164 [-.305, -.023] | -.003 [-.128, .122] | |
| 7. Hope | -- | .792 [.723, .862] | -.118 [-.269, .033] | -.309 [-.454, -.164] | -.045 [-.177, .088] | .032 [-.101, .164] | |
| 8. Purpose |  | -- | -.089 [-.251, .074] | -.261 [-.400, -.121] | -.061 [-.199, .077] | -.081 [-.069, .230] | |
| 9. Somatic Complaints |  |  | -- | .818 [.695, .941] | .056 [-.101, .212] | -.108 [-.264, .047] | |
| 10. Psychological Complaints |  |  |  | -- | .130 [-.019, .279] | .014 [-.133, .161] | |
| 11. Substance Abuse |  |  |  |  | -- | .370 [.181, .559] | |
| 12. Antisocial Behavior |  |  |  |  |  | -- | |
|  |  |  |  |  |  |  | |
